# Tolvaptan add‐on therapy in patients with acute heart failure: A systematic review and meta‐analysis

**DOI:** 10.1002/prp2.614

**Published:** 2020-06-04

**Authors:** Xiandu Luo, Qi Jin, Yanqing Wu

**Affiliations:** ^1^ Department of Cardiovascular Medicine The Second Affiliated Hospital of Nanchang University Nanchang Jiangxi People’s Republic of China; ^2^ Center for Pulmonary Vascular Diseases National Center for Cardiovascular Diseases Fuwai Hospital Chinese Academy of Medical Sciences and Peking Union Medical College Beijing People’s Republic of China

**Keywords:** acute heart failure, meta‐analysis, tolvaptan, traditional diuretics

## Abstract

This study aimed to investigate the short‐term efficacy and safety of tolvaptan as an add‐on to traditional diuretics in patients with acute heart failure (AHF). The PubMed, EMBASE, Cochrane Library, and Web of Science databases were comprehensively searched for all randomized controlled trials (RCTs) that examined AHF patients treated with tolvaptan as a combination therapy with traditional diuretics published on or before December 2, 2019. Efficacy indicators such as improved dyspnea, reduced edema, and changes in urine output and body weight were evaluated. In‐hospital mortality and worsening renal function (WRF) were measured as safety indicators. Data from the published literature included in this study were independently extracted by two reviewers. The Cochrane risk of bias tool was used to evaluate the quality of the included RCTs. Twelve RCTs involving 5577 patients admitted for AHF were included. Compared with traditional diuretics alone, add‐on tolvaptan significantly relieved dyspnea, reduced weight, increased total urine volume and changes in urine volume from baseline, reduced edema, and increased serum sodium concentration in the short term without increasing the mortality. Most importantly, a low dose of tolvaptan (7.5‐15 mg/d) significantly reduced the incidence of WRF, while a high dose (30 mg/d) had the opposite effect. Short‐term add‐on tolvaptan in hospitalized AHF patients could significantly relieve shortness of breath, reduce body weight, improve edema, and increase urine output and serum sodium concentrations without increasing mortality. The protective effects of add‐on tolvaptan against WRF, however, were observed at low doses, but not at high doses.

**Abbreviations**
AHF: acute heart failure
AVP: arginine vasopressin
CI: confidence interval
MD: mean difference
RAAS: renin‐angiotensin‐aldosterone system
RCTs: randomized controlled trials
RR: relative risk
SNS: sympathetic nervous system
WRF: worsening renal function

## 1 INTRODUCTION

Fluid retention is the main cause of the signs and symptoms patients with acute heart failure (AHF) experience, and diuretic therapy is currently the only pharmacological treatment that promotes fluid excretion. Approximately 80% of hospitalized patients with AHF require intravenous diuretics, demonstrating the cornerstone role of diuretics in this patient population.^1^ Traditional diuretics include thiazides, potassium‐sparing diuretics, and loop diuretics, the latter of which have become first‐line agents for AHF through their inhibition of the reabsorption of chloride and sodium ions in the ascending loop of Henle.^2^ However, about one third of patients with heart failure experience diuretic resistance, that is, the standard dose of diuretics does not achieve ideal urine output or effectively relieve congestion.^3^ Although this may be ameliorated by increasing the dose or by adding thiazide diuretics, this could activate the renin‐angiotensin‐aldosterone system (RAAS) and increase the risk of electrolyte imbalance, renal dysfunction, and in‐hospital mortality.^4^, ^5^, ^6^

Tolvaptan is a nonpeptide, selective vasopressin V_2_ receptor antagonist that exerts a diuretic effect by binding to and blocking the activity of vasopressin V_2_ receptors, lowering the expression of aquaporin AQP2 on collecting duct intimal cells, and reducing water reabsorption without affecting the absorption and excretion of sodium and potassium ions.^7^ Currently, the efficacy and safety of tolvaptan in the treatment of heart failure remains controversial. Studies have shown that tolvaptan added to traditional diuretics can significantly increase urine volume without causing electrolyte disturbances, while others have demonstrated that add‐on tolvaptan was not superior to conventional diuretics alone in improving the congestive symptoms of heart failure, and there was a risk of worsening renal function (WRF).^8^, ^9^, ^10^ A meta‐analysis of the short‐term (≤7 days) efficacy and safety of tolvaptan in AHF patients found that tolvaptan did not reduce the incidence of WRF or short‐term all‐cause mortality.^11^ However, only a limited number of randomized controlled trials (RCTs) were included in that study, and the Efficacy of Vasopressin Antagonism in Heart Failure Outcome Study with Tolvaptan (EVEREST), a pivotal study with the largest sample size, was not included, possibly causing a considerable bias. Therefore, it is necessary to re‐evaluate the short‐term efficacy and safety of add‐on tolvaptan in AHF patients.

## 2 MATERIALS AND METHODS

### 2.1 Literature search

**Figure 1:**
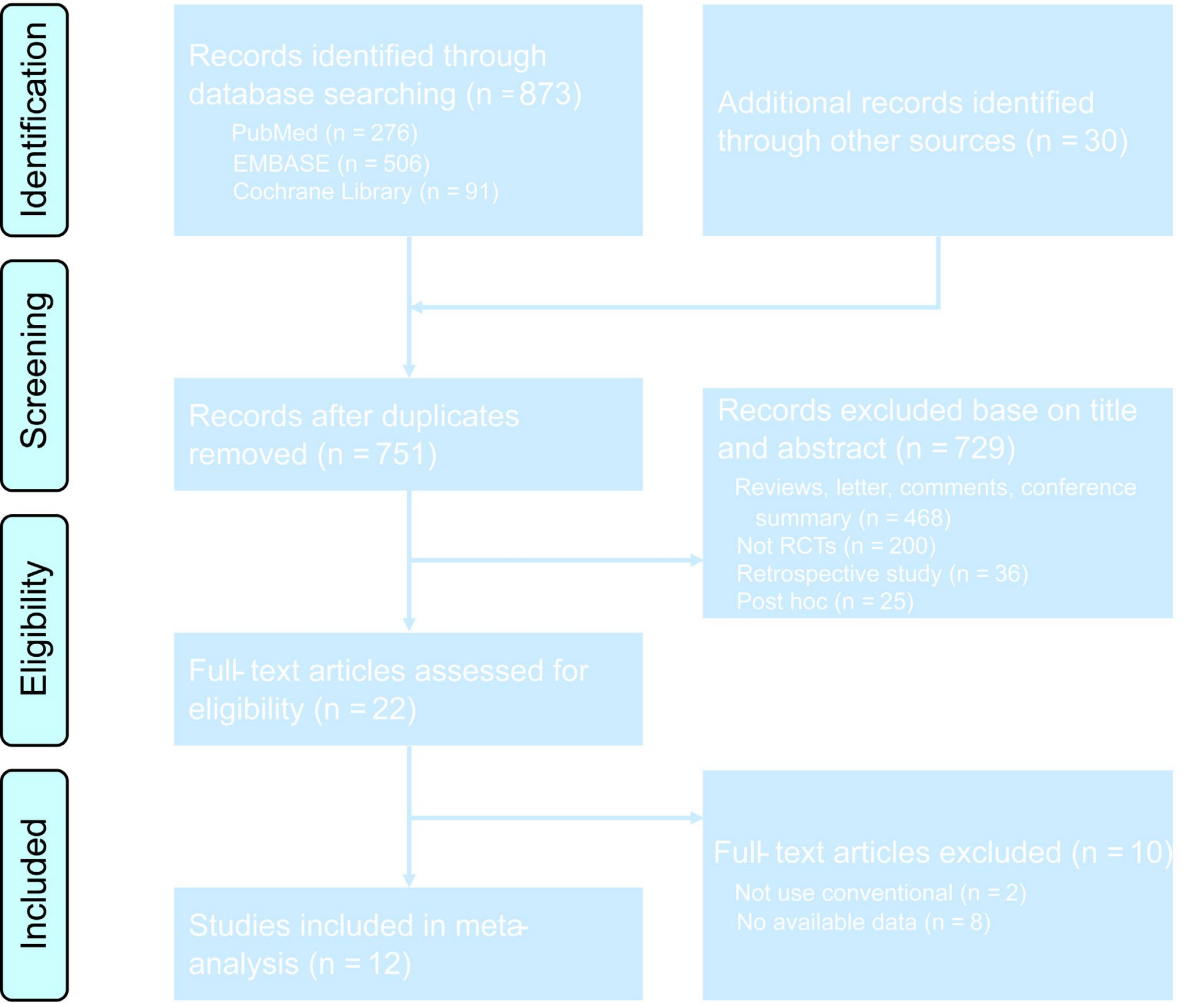
Flow diagram of the study selection process for the meta‐analysis

The PubMed, EMBASE, Cochrane Library, and Web of Science databases were systematically searched for RCTs involving tolvaptan in the treatment of heart failure up to December 2, 2019. The literature selection, data extraction, and quality assessment of the included studies were conducted by two independent reviewers (XDL and QJ). Any discordance was organized, investigated, and resolved by the senior author. The search strategy included MeSH terms and the keywords “tolvaptan”, “heart failure”, and “RCT”. Detailed search formulas are provided in Tables S1 and S2. The meta‐analysis was reported according to the PRISMA Statement (Figure 1; Data S1).^12^

### 2.2 Study selection

This meta‐analysis included only RCTs of hospitalized patients with AHF, where tolvaptan was an add‐on to traditional diuretics in the treatment group, and traditional diuretics were compared in the control group. Reviews, editorials, case reports, conference summaries, medical reports, and retrospective analyses from the same clinical trials were excluded. An email was sent to the authors to obtain additional information on eligible papers with insufficient information, and studies were excluded if no related data were provided.

### 2.3 Data extraction and quality assessment

The basic information extracted from the RCTs included: the corresponding author, publication date, sample size, patient characteristics, trial design, follow‐up time, and region. Efficacy indicators included relief of dyspnea; reduction in edema; and changes in body weight, urine output, and serum sodium concentration from the initiation of medication therapy until discharge. Patient safety indicators included in‐hospital mortality and WRF. The quality of the included studies was evaluated using the Cochrane risk of bias tool in the Review Manager.^13^

### 2.4 Statistical analysis

Data were analyzed using the Review Manager 5.3, and relative risk (RR), mean difference (MD), and the 95% confidence interval (CI) were selected as effect indicators. To determine whether significant heterogeneity existed, the χ^2^ and *I* ^2^ tests were evaluated first, and the fixed‐effect model was used for analysis when nonsignificant heterogeneity was indicated (*P* > .1 and *I* ^2^ < 50%). A random‐effects model was used when *P* < .1 and *I* ^2^ ≥ 50%, and further subgroup analyses were performed to explore the possible source of statistical heterogeneity. The purpose of the sensitivity analysis was to test the stability of our results by removing each study individually and recalculating the results to determine whether our estimates were affected by a particular study. Differences were considered statistically significant when *P* < .05.

## 3 RESULTS

### 3.1 Eligible studies

**Figure 2:**
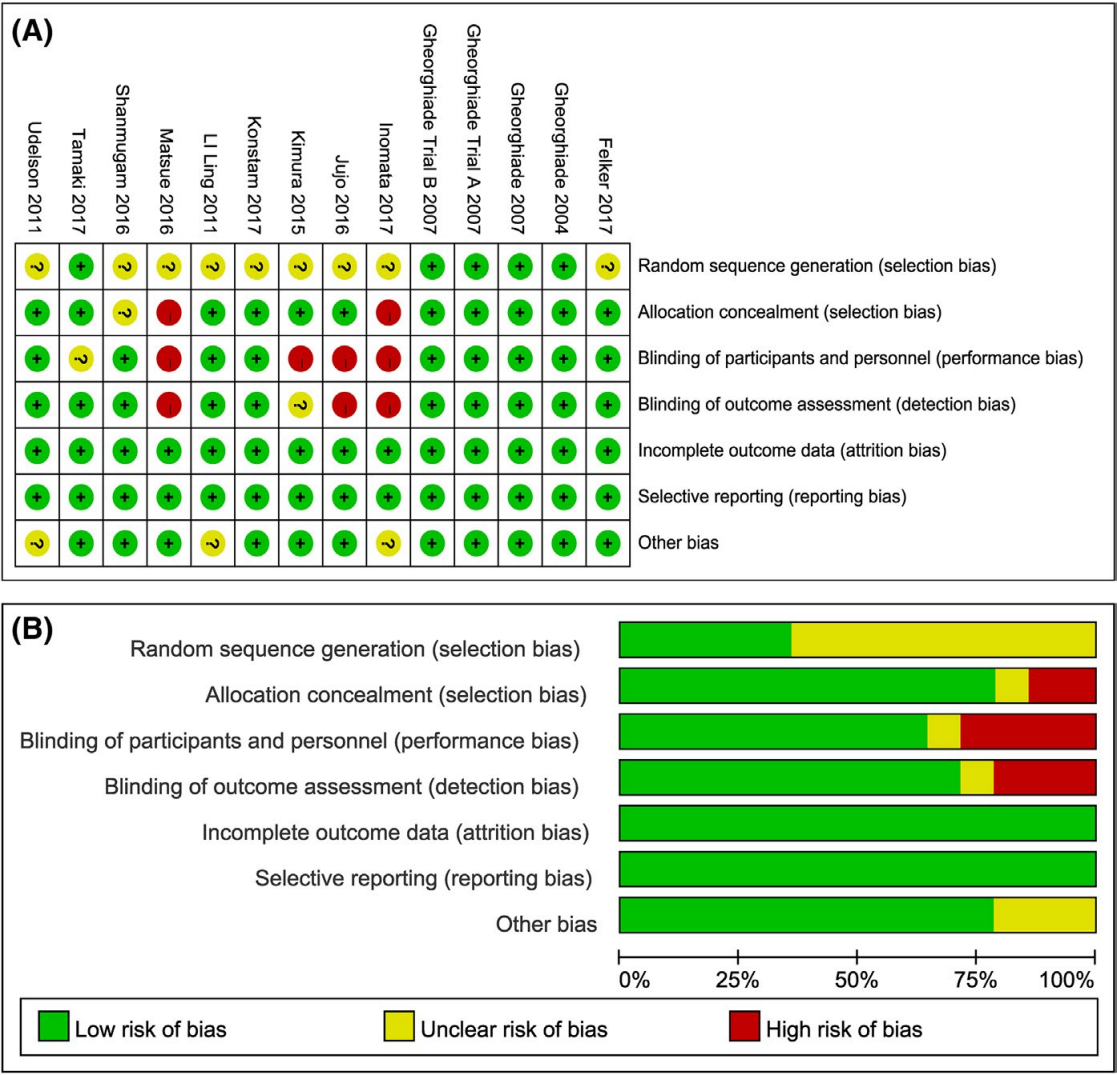
The quality of included RCTs: A, risk of bias per item for each included RCT; B, risk of bias per item presented as percentages across all included RCTs

A total of 903 articles were retrieved based on the established search strategy. A total of 5577 hospitalized patients with heart failure from 12 articles, including 13 clinical trials (EVEREST was divided into Trial A and Trial B), were included after the inclusion and exclusion criteria were assessed.^10^, ^14^, ^15^, ^16^, ^17^, ^18^, ^19^, ^20^, ^21^, ^22^, ^23^, ^24^ The basic characteristics of the included trials are shown in Table 1. There was one open‐label, four single‐blind, and seven double‐blind RCTs. Four were from North America, and seven from Asia. The remaining EVEREST study was a global multicentre RCT. The Cochrane risk of bias tool was used to evaluate the quality of the included RCTs (Figure 2). Although some studies had insufficient information about individual items, most items within the tool were considered low risk, indicating that the RCTs included in the meta‐analysis were relatively high quality.

**Table 1:** Basic characteristics of included clinical trials

Author, date	Clinical trial no.	Acronym	Patient characteristics	Trial design	Intervention		Sample size	Follow‐up	Study location
					Tolvaptan	Control			
Gheorghiade 2004	NR	ACTIV in CHF	CHF	Mul, Ran, DB, PCtr	TLV 30 mg/d + Con	Pla + Con	320	7 d	Argentina, USA
Gheorghiade 2007	NCT00071331	EVEREST	CHF	Mul, Ran, DB, PCtr	TLV 30 mg/d + Con	Pla + Con	4133	7 d	18 Countries
Udelson 2011	NR	NR	HF	Mul, Ran, DB, PCtr	TLV 30 mg/d + FUR	Pla + FUR	41	8 d	USA
LI Ling 2011	NR	NR	HF	Mul, Ran, DB, PCtr	TLV 15‐60 mg/d + Con	Pla + Con	65	7 d	China
Matsue 2016	UMIN000007109	AQUAMARINE	AHF, RI	Mul, Ran, OL, Ctr	TLV 15 mg/d + FUR	FUR	217	2 d	Japan
Shanmugam 2016	CTRI/2013/05/003643	NR	AHF, Hyp	Ran, DB, PCtr	TLV 15 mg/d + Con	Pla + Con	51	5 d	India
Jujo 2016	UMIN000014134	NR	CHF	Ran, OL, Ctr	TLV 7.5 mg/d + Car	FUR + Car	60	5 d	Japan
Kimura 2016	NR	TACT‐ADHF	ADHF	Ran, SB, Ctr	TLV 15 mg/d + FUR	FUR	52	7 d	Japan
Tamaki 2017	UMIN000013727	NR	ADHF	Ran, OL, Ctr	TLV 7.5‐15 mg/d + Con	Con	50	48 h	Japan
Inomata 2017	UMIN000009201	K‐STAR	HF, RI	Mul, Ran, OL, Ctr	TLV 7.5‐15 mg/d + Con	Con	81	7 d	Japan
Konstam 2017	NCT01584557	SECRET of CHF	HF, RI	Mul, Ran, DB, PCtr	TLV 30 mg/d + Con	Pla + Con	250	7 d	USA
Felker 2017	NCT01644331	TACTICS‐HF	HF	Mul, Ran, DB, PCtr	TLV 30 mg/d + Con	Pla + Con	257	48 h	USA

Abbreviations: ADHF, acute decompensated heart failure; AHF, acute heart failure; Car, carperitide; CHF, congestive heart failure; Ctr, controlled; Con, conventional diuretic; DB, double‐blind; FUR, furosemide; HF, heart failure; Hyp, hyponatremia; Mul, multicentre; NR, not reported; OL, open‐labeled; Pla, placebo; PCtr, placebo‐controlled; Ran, randomized; RI, renal impairment; SB, single‐blind; TLV, tolvaptan.

### 3.2 Efficacy evaluation

**Figure 3:**
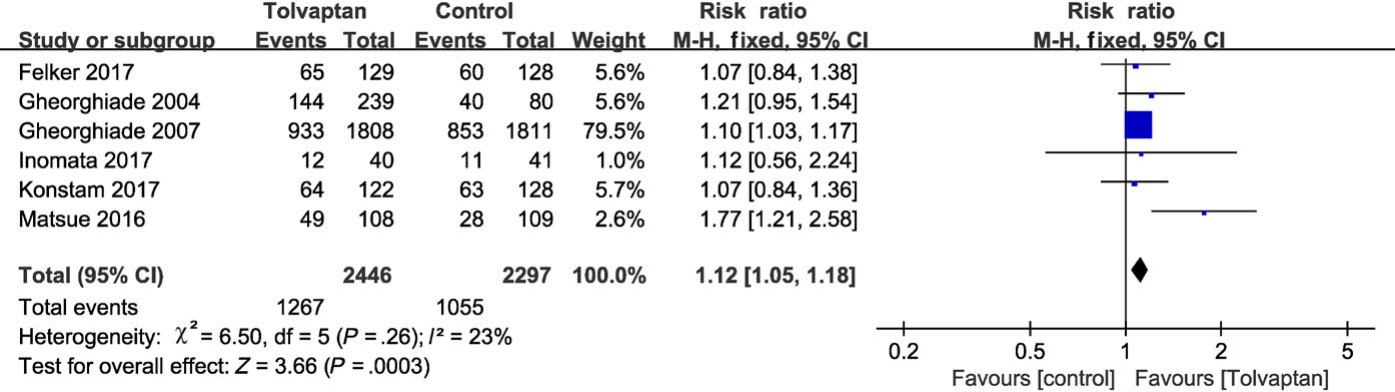
Forest plot depicting the effects of tolvaptan on dyspnea: tolvaptan was more effective in relieving dyspnea

**Figure 4:**
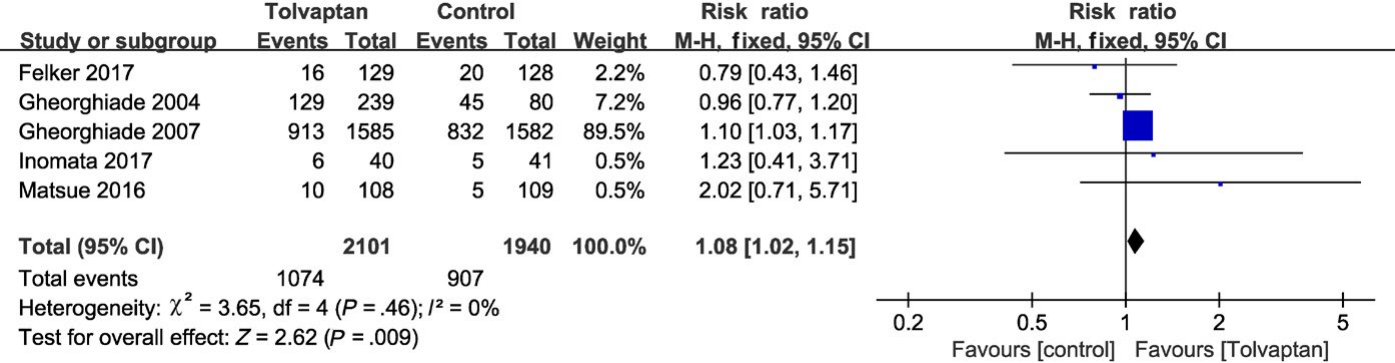
Forest plot depicting the effects of tolvaptan on edema: tolvaptan significantly reduced edema

**Figure 5:**
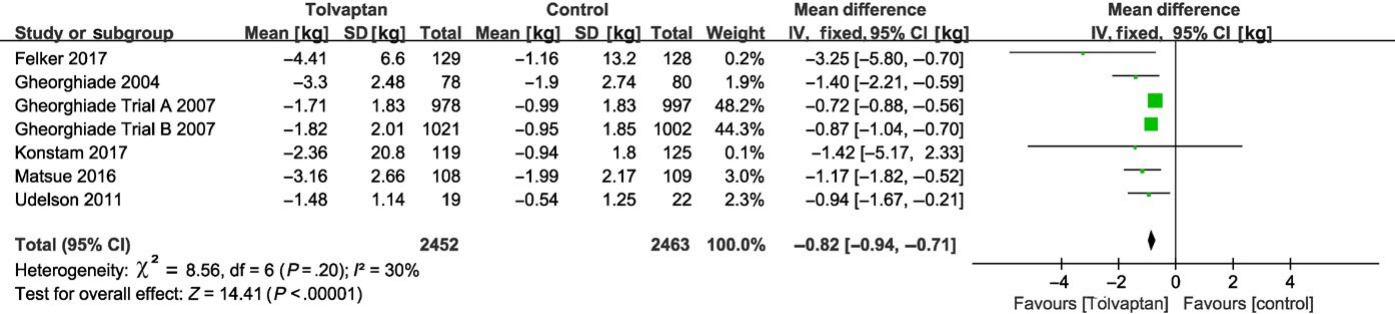
Forest plot depicting the effects of tolvaptan on body weight: tolvaptan clearly reduced body weight

**Figure 6:**
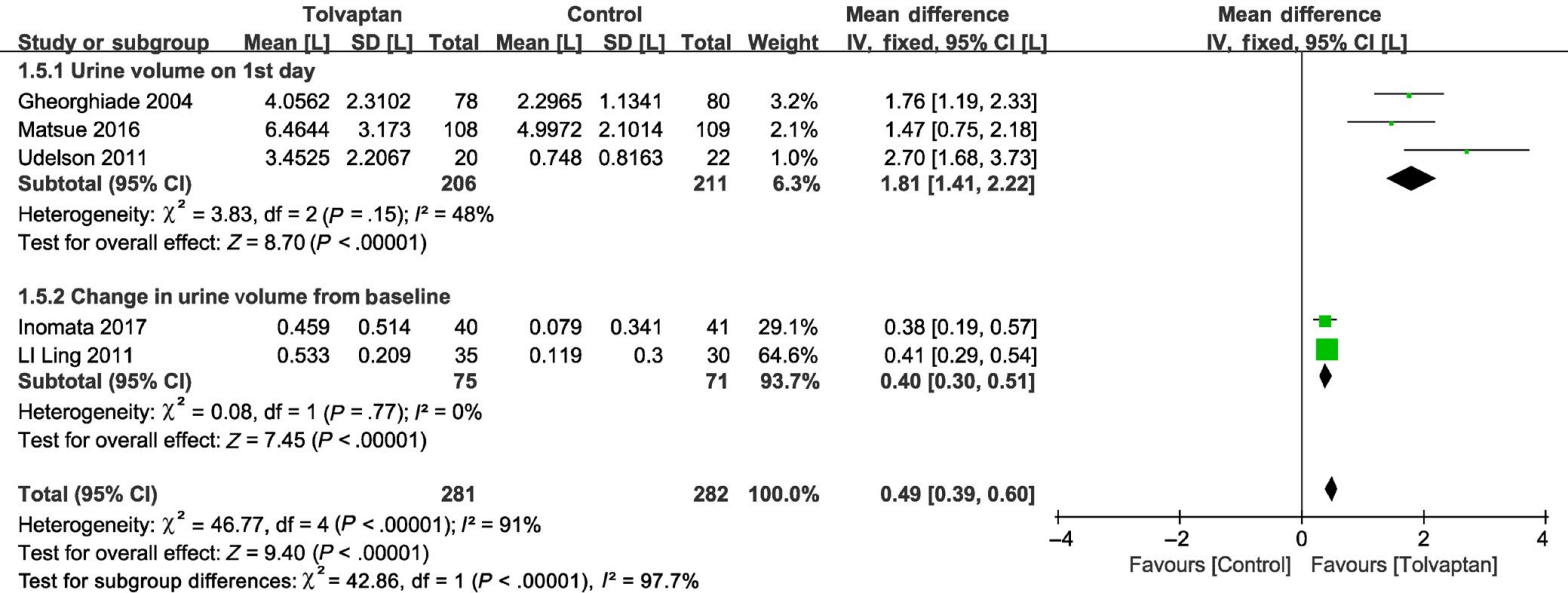
Forest plot depicting the effects of tolvaptan on urine volume: tolvaptan was better than traditional diuretics alone at increasing urine output

**Figure 7:**
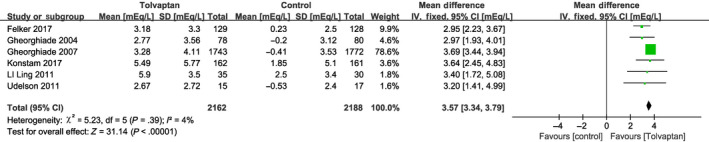
Forest plot depicting the effects of tolvaptan on serum sodium concentration: tolvaptan could increase serum sodium concentrations

Dyspnea was one of the main reasons for the admission of AHF patients to the hospital, and a total of six studies reported the number of patients who had relief of dyspnea on the second hospital day.^10^, ^19^, ^21^, ^22^, ^23^, ^24^ No significant heterogeneity was observed between these studies (*P* = .26, *I* ^2^ = 23%; Figure 3), thus the analysis was performed using the fixed‐effect model and add‐on tolvaptan was shown to be more effective in relieving short‐term dyspnea than traditional diuretics alone (RR = 1.12, 95% CI [1.05‐1.18], *P* < .001). Reduced urine output and excessive fluid retention in patients with heart failure often meant they presented with pitting edema and nonnutritional weight gain, which could be improved with diuretics. Five studies reported reduced edema with diuretics after admission.^10^, ^19^, ^22^, ^23^, ^24^ Since no significant heterogeneity was observed among the studies, the fixed‐effect model was used for the analysis (*P* = .46, *I* ^2^ = 0%). According to these studies, tolvaptan add‐on therapy was more effective at reducing edema than conventional diuretics alone (RR = 1.08, 95% CI [1.02‐1.15], *P* = .009; Figure 4). Body weight changes over 24 hours were discussed in seven clinical trials from six studies (EVEREST was divided into Trial A and Trial B),^10^, ^18^, ^19^, ^21^, ^23^, ^24^ and the fixed‐effect model was used since no significant heterogeneity existed (*P* = .20, *I* ^2^ = 30%). Short‐term loss of body weight was more pronounced in the add‐on tolvaptan group than in the conventional diuretics group (MD = −0.82, 95% CI [−0.94 to 0.71], *P* < .001; Figure 5). As a result of the significant heterogeneity found among the five studies that discussed urine volume, however, a subgroup analysis was conducted (*P* < .001, *I* ^2^ = 91%; Figure 6).^18^, ^19^, ^20^, ^22^, ^24^ There was no significant heterogeneity among the three studies reporting first‐day urine volumes (*P* = .15, *I* ^2^ = 48%) or among the two studies that discussed urine volume changes from baseline (*P* = .77, *I* ^2^ = 0%). In these studies, add‐on tolvaptan was found to be superior to traditional diuretics alone at increasing urine output (Figure 6). In addition, short‐term serum sodium concentration was higher in the add‐on tolvaptan group than the traditional diuretics‐alone group (MD = 3.57, 95% CI [3.34‐3.79], *P* < .001; Figure 7).^10^, ^18^, ^20^, ^21^, ^23^, ^24^

### 3.3 Safety evaluation

**Figure 8:**
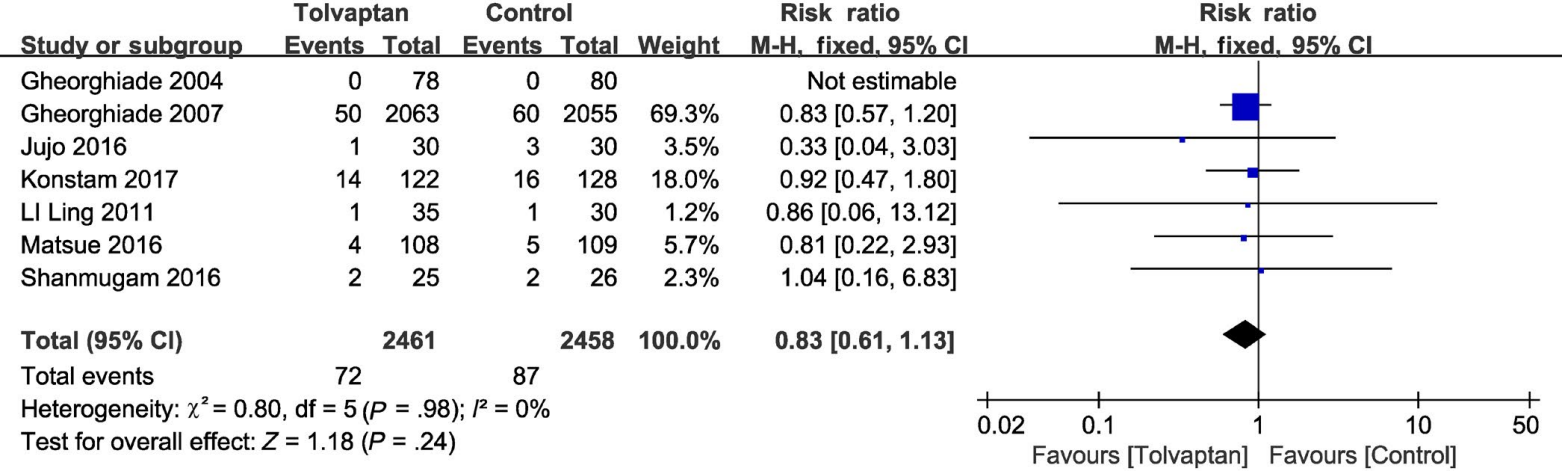
Forest plot depicting the effects of tolvaptan on mortality: tolvaptan did not increase mortality during hospitalization more than traditional diuretics alone

**Figure 9:**
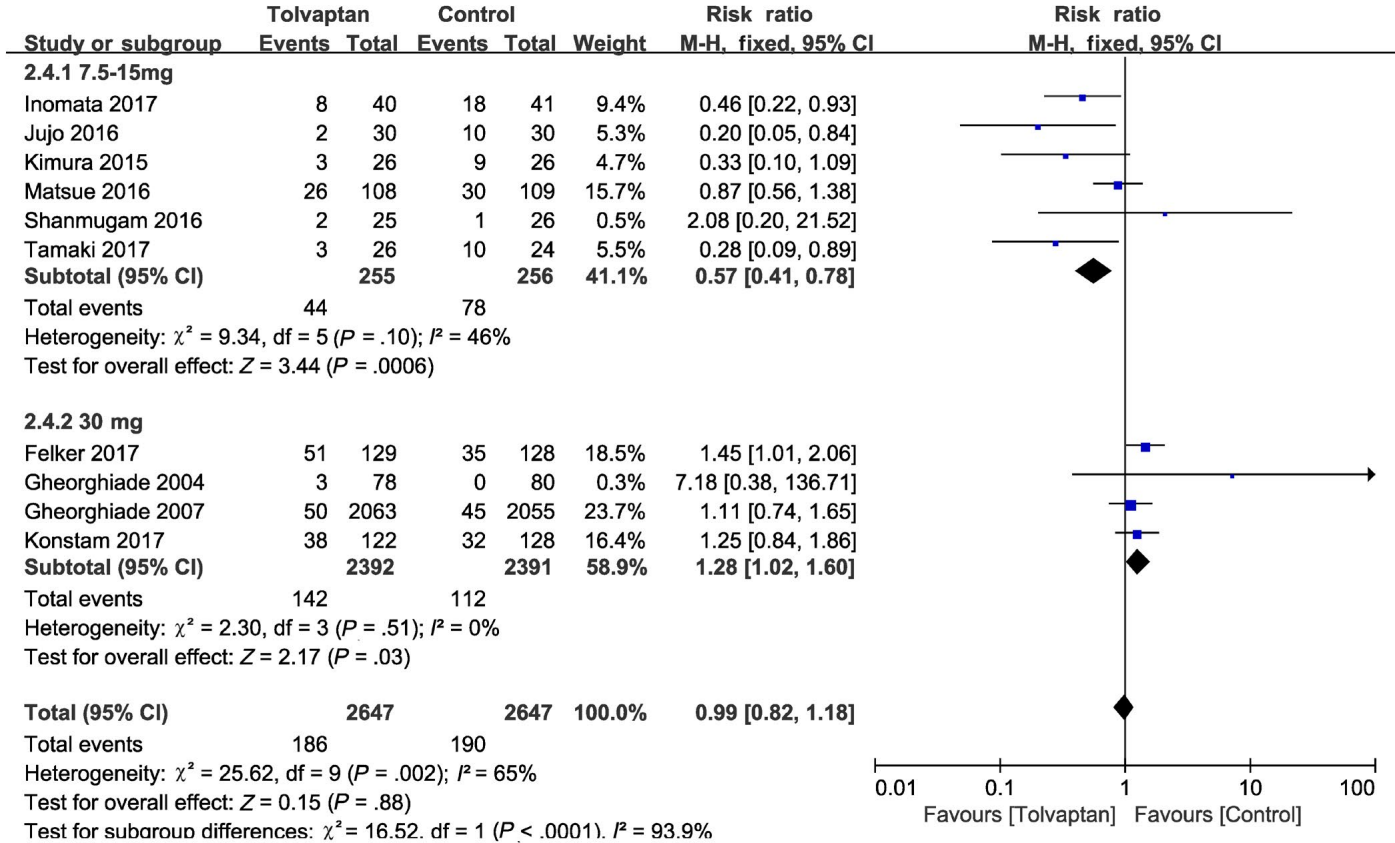
Forest plot depicting the effects of tolvaptan on worsening renal function (WRF): The incidence of WRF was associated with the dose of tolvaptan; a low dose of tolvaptan could significantly reduce the incidence of WRF, while a high dose did the opposite

There was no significant heterogeneity observed among the five RCTs that reported mortality (*P* = .98, *I* ^2^ = 0%; Figure 8),^15^, ^17^, ^19^, ^20^, ^21^, ^23^, ^24^ and add‐on tolvaptan was not found to increase in‐hospital mortality compared with conventional diuretics (RR = 0.83, 95% CI [0.61‐1.13], *P* = .24). WRF was defined as a 0.3 mg/dL increase in serum creatinine from baseline after randomization. Significant heterogeneity was identified among 10 RCTs reporting changes in renal function during hospitalization (*P* = .002, *I* ^2^ = 65%; Figure 9), and the results of the random‐effects model showed that add‐on tolvaptan did not reduce the incidence of WRF during hospitalization in AHF patients. A subgroup analysis was performed after dividing these studies into a low‐dose (7.5‐15 mg/d) tolvaptan group^14^, ^15^, ^16^, ^17^, ^19^, ^22^ and a high‐dose tolvaptan (30 mg/d) group.^10^, ^21^, ^23^, ^24^ For these groups, there was no significant heterogeneity (*P* = .10, *I* ^2^ = 46% and *P* = .51, *I* ^2^ = 0%, respectively), thus the fixed‐effect model was chosen for further analysis. Low‐dose tolvaptan add‐on therapy was found to significantly reduce the incidence of WRF (RR = 0.57, 95% CI [0.41‐0.78], *P* < .001), whereas high‐dose tolvaptan had the opposite effect (RR = 1.28, 95% CI [1.02‐1.60], *P* = .03).

## 4 DISCUSSION

Our meta‐analysis demonstrated that add‐on tolvaptan could significantly alleviate the signs and symptoms of volume overload and increase the serum sodium concentration in the short term without increasing mortality. Subgroup analysis suggested that low‐dose tolvaptan add‐on therapy could significantly reduce the incidence of WRF, whereas the opposite was shown in the high‐dose group.

Most AHF patients are admitted to the hospital with dyspnea, edema, and weight gain caused by volume overload. Therefore, using diuretics to rapidly decrease volume load is essential for heart failure treatment. Consistent with previous studies,^10^, ^25^ this meta‐analysis found that add‐on tolvaptan could significantly reduce volume overload as evidenced by relieved dyspnea, reduced weight, and increased total urine volume and changes in urine volume from baseline.

Electrolyte disturbances such as hyponatremia (serum sodium concentration <135 mmol/L) induced by diuretics are common among patients with heart failure.^26^ The rapid correction of hyponatremia could significantly shorten hospital stays and reduce cognitive impairment caused by neurological disorders.^27^ Isotonic saline can easily correct hypovolemic hyponatremia but not nonhypovolemic hyponatremia.^28^ Improvement in hyponatremia has been seen as a benefit of tolvaptan over traditional diuretics.^29^, ^30^ Although both our study and the EVEREST study reported that add‐on tolvaptan could increase the incidence of hypernatremia, excessive levels of sodium did not require additional intervention.^23^ Therefore, the short‐term benefit of tolvaptan as an add‐on to traditional diuretics among patients with AHF was definite, although an increased sodium concentration within an acceptable range might be encountered.

The EVEREST long‐term results suggested that add‐on tolvaptan did not increase all‐cause mortality.^10^, ^23^, ^24^ Similarly, our meta‐analysis demonstrated that adding tolvaptan to traditional diuretics did not increase in‐hospital mortality. Patients with AHF often have decreased renal perfusion due to circulatory hypovolemia.^14^ Traditional diuretics can rapidly improve congestion, but can result in reduced blood volume and progressive renal dysfunction,^31^ which could further activate the RAAS and sympathetic nervous system (SNS), two important pathophysiological mechanisms of ventricular remodeling and impaired renal function.^32^, ^33^ For instance, loop diuretics can activate RAAS by reducing sodium concentration near the macula densa.^34^ Thus, traditional diuretics are thought to lead to WRF. In contrast to traditional diuretics such as loop diuretics, tolvaptan blocked the reabsorption of urea, reduced serum urea nitrogen,^23^ and possessed a weaker ability to activate SNS and RAAS.^17^ Therefore, there are high hopes for tolvaptan, but whether tolvaptan can reduce renal damage has been debated. The Acute and Chronic Therapeutic Impact of a Vasopressin Antagonist in Chronic Heart Failure study found that tolvaptan tended to protect renal function in patients with AHF, but the EVEREST study, with the largest sample size, demonstrated a slight increase in serum creatinine.^23^, ^24^ Interestingly, even with more clinical trials published, this controversy has not been resolved. In the TACTICS study, for example, add‐on tolvaptan increased WRF in AHF patients,^10^ whereas the K‐STAR study showed a significant increase in urine volume and improvement in renal function.^22^

In this meta‐analysis, low‐dose tolvaptan add‐on therapy was correlated with a lower incidence of WRF than traditional diuretics alone, which may be due to its protective role in renal function as described above. In addition, some researchers believe that improved WRF might be attributed to the lower loop diuretic dose as a result of add‐on tolvaptan,^35^ since a positive correlation between WRF and loop diuretic dose has previously been observed.^36^ Unfortunately, we could not analyze this further since no accurate loop diuretic doses were provided in any of the included studies. However, the incidence of WRF was higher in AHF patients receiving high‐dose tolvaptan add‐on therapy than in patients receiving traditional diuretics, although the reason for this was unclear. Additionally, patients with heart failure were sensitive to blood sodium concentrations, with even normal levels stimulating the release of arginine vasopressin (AVP). A single‐centre RCT found that a single dose of 15‐120 mg of tolvaptan showed a dose‐dependent blood sodium concentration,^37^ and high doses of tolvaptan might profoundly increase blood sodium concentration, leading to release and subsequent binding of AVP to the V_1_a receptor to cause vasoconstriction and renal hypoperfusion. Conivaptan, a dual V_1_a and V2 receptor antagonist, was able to prevent renal damage in rats that ingested large amounts of hypertonic fructose.^38^ Compared with tolvaptan, conivaptan had a lower chance of causing hypernatremia,^39^ indicating that V_1_a and V_2_ dual receptor inhibitors might be a potential shining star for acute heart failure therapy.

This study has several limitations. First, some included studies were single blind or open label, which might have resulted in biases. Second, it was impossible to determine the role of traditional diuretics, especially loop diuretics, in the WRF subgroup analysis because their administration and dosage were not accurately reported. Third, the EVEREST study accounted for 80% of the enrolled population, therefore, the conclusions were biased toward the conclusions of the EVEREST study. Finally, the included RCTs had obvious geographical disparity, since most studies were from North America and Japan, and the studies from Japan were supplemented with low‐dose tolvaptan, while North American studies used high doses. Therefore, extending the conclusions drawn from dose analysis to other regions or races should be done with caution. High‐quality RCTs involving different races and doses are needed to confirm these results.

## 5 CONCLUSION

Compared with traditional diuretics, short‐term add‐on tolvaptan in hospitalized AHF patients could significantly relieve dyspnea, increase urine output, reduce body weight and edema, and increase serum sodium concentration, without increasing mortality. Importantly, the protective effect of tolvaptan add‐on therapy against WRF was observed at low doses, but not at high doses.

## CONFLICT OF INTEREST

The authors declare that they have no competing interests.

## AUTHOR CONTRIBUTIONS

XDL and QJ searched the literature, extracted data from the collected literature, analyzed the data, and wrote the manuscript. YQW made substantial contributions to the conception and design of the study and revised the manuscript. All authors approved the final version of the manuscript.

## Supporting information

Supinfo S1

Supinfo S2

## Data Availability

All data generated and analyzed in the study are available from the corresponding author upon reasonable request.
