# Supplementary material for: Tolvaptan add‐on therapy in patients with acute heart failure: A systematic review and meta‐analysis
Source: Pharmacol Res Perspect. 2020 Jun 4;8(3):e00614. doi: 10.1002/prp2.614 (PMC7272393; doi:10.1002/prp2.614)
Supplement: Supplementary file 1 — Supinfo S1 [file PRP2-8-e00614-s001.docx]

**Additional file 1: Table A.** Search strategy in PubMed

| Recent queries in PubMed | |  |
| --- | --- | --- |
| Search | Query | Items found |
| #1 | Search (((("Heart Failure"[Mesh]) OR ((((((((((((((Cardiac Failure[Title/Abstract]) OR Heart Decompensation[Title/Abstract]) OR Decompensation, Heart[Title/Abstract]) OR Heart Failure, Right-Sided[Title/Abstract]) OR Heart Failure, Right Sided[Title/Abstract]  ) OR Right-Sided Heart Failure[Title/Abstract]) OR Right Sided Heart Failure[Title/Abstract]) OR Myocardial Failure[Title/Abstract]) OR Congestive Heart Failure[Title/Abstract]) OR Heart Failure, Congestive[Title/Abstract]) OR Heart Failure, Left-Sided[Title/Abstract]) OR Heart Failure, Left Sided[Title/Abstract]) OR Left-Sided Heart Failure[Title/Abstract]) OR Left Sided Heart Failure[Title/Abstract]))) AND (("Tolvaptan"[Mesh]) OR (((((7-Chloro-5-hydroxy-1- AND (2-methyl-4- AND (2-methylbenzoylamino) AND benzoyl) AND 2,3,4,5-tetrahydro-1H-1-benzazepine[Title/Abstract]) OR Samsca[Title/Abstract]) OR OPC 41061[Title/Abstract]) OR OPC-41061[Title/Abstract]) OR OPC41061[Title/Abstract]))) AND (randomized controlled trial[pt] OR controlled clinical trial[pt] OR randomized[tiab] OR placebo[tiab] OR drug therapy[sh] OR randomly[tiab] OR trial[tiab] OR groups[tiab] NOT (animals[mh] NOT humans[mh])) | 276 |

**Additional file 1: Table B.** Search strategy in EMBASE

| No. | Query Results | Results |
| --- | --- | --- |
| #23 | #21 AND #22 | 506 |
| #22 | 'crossover procedure':de OR 'double-blind procedure':de OR 'randomized controlled trial':de OR 'single-blind procedure':de OR random*:de,ab,ti OR factorial*:de,ab,ti OR crossover*:de,ab,ti OR ((cross NEXT/1 over*):de,ab,ti) OR placebo*:de,ab,ti OR ((doubl* NEAR/1 blind*):de,ab,ti) OR ((singl* NEAR/1 blind*):de,ab,ti) OR assign*:de,ab,ti OR allocat*:de,ab,ti OR volunteer*:de,ab,ti | 24822926 |
| #21 | #7 AND #20 | 1253 |
| #20 | #8 OR #9 OR #10 OR #11 OR #12 OR #13 OR #14 OR #15 OR #16 OR #17 OR #18 OR #19 | 509687 |
| #19 | 'decompensation, heart':ab,ti | 15 |
| #18 | 'right-sided heart failure':ab,ti | 946 |
| #17 | 'heart failure, congestive':ab,ti | 53 |
| #16 | 'heart failure, left-sided':ab,ti | 4 |
| #15 | 'heart failure, left sided':ab,ti | 4 |
| #14 | 'left sided heart failure':ab,ti | 329 |
| #13 | 'congestive heart failure':ab,ti | 52333 |
| #12 | 'myocardial failure':ab,ti | 936 |
| #11 | 'right sided heart failure':ab,ti | 946 |
| #10 | 'heart decompensation':ab,ti | 142 |
| #9 | 'cardiac failure':ab,ti | 16918 |
| #8 | 'heart failure'/exp | 493553 |
| #7 | #1 OR #2 OR #3 OR #4 OR #5 OR #6 | 2546 |
| #6 | 'opc41061':ti,ab | 3 |
| #5 | 'opc-41061':ti,ab | 16 |
| #4 | opc 41061':ti,ab | 16 |
| #3 | 'samsca':ti,ab | 13 |
| #2 | '7-chloro-5-hydroxy-1-(2-methyl-4-(2-methylbenzoylamino)benzoyl)2,3,4,5-tetrahydro-1h- 1-benzazepine':ti,ab | 1 |
| #1 | 'tolvaptan'/exp OR 'tolvaptan' | 2,546 |
